# Thermal Sensing of Photo-Activated Dental Resin Composites Using Infrared Thermography

**DOI:** 10.3390/polym15204117

**Published:** 2023-10-17

**Authors:** Turki A. Bakhsh, Abdulaziz Alfaifi, Yousef Alghamdi, Mohannad Nassar, Roaa A. Abuljadyel

**Affiliations:** 1 Restorative Dentistry Department, Faculty of Dentistry, King Abdulaziz University, P.O. Box 80209, Jeddah 215-89, Saudi Arabia; rabuljadaiel@kau.edu.sa; 2 Cariology and Operative Dentistry, Tokyo Medical and Dental University, Tokyo 113-8549, Japan; 3 Advanced Technology Dental Research Laboratory, Faculty of Dentistry, King Abdulaziz University, P.O. Box 80209, Jeddah 215-89, Saudi Arabia; dentazizt@gmail.com (A.A.); yousef1789@gmail.com (Y.A.); 4 Department of Preventive and Restorative Dentistry, College of Dental Medicine, University of Sharjah, Sharjah P.O. Box 27272, United Arab Emirates; minassar@sharjah.ac.ae

**Keywords:** camera, thermal, pulp, composite, polymerization, temperature

## Abstract

Objective: The goal of this study was to compare the pulp temperature increase during light curing of different composite thicknesses in deep class I cavities using two thermal sensing tools. Methodology: Round occlusal class I cavities with a remaining dentin thickness (RDT) of 1 mm from the cavity floor were performed on 15 extracted sound molars. Samples were divided into three groups (*n* = 5). In group A, cavities were restored using the Filtek Z350 XT conventional composite through the incremental filling technique, whereas group B cavities were restored using the Filtek Bulk-Fill flowable composite through the bulk-fill technique. Specimens of the Filtek Bulk-Fill flowable composite using the incremental filling technique were used to restore cavities in group C. An infrared thermal camera (IRT; Flir, Wilsonville, OR, USA) and K-type thermocouple (Perfect Prime TC0520, New York, NY, USA) were used to measure the heat generated during composite photo-polymerization. Results: There were no significant differences within the same group using either the thermocouple or IRT (*p* > 0.05). One-way ANOVA showed no significant differences between groups A and C (*p* > 0.05), whereas group B was significantly different from groups A and C with each sensing tool (*p* < 0.05). Conclusion: IRT and thermocouple heat readings were comparable. Based on the current findings, the bulk-fill technique resulted in the lowest heat generation among the groups. Therefore, increasing the light-curing time and the number of composite increments was directly correlated with increases in intra-pulpal temperature.

## 1. Introduction

Various dental treatments require different materials and instruments that may generate heat that could have an influence on pulpal health. The use of a high-speed handpiece increases temperature due to friction [1]. Several other causes, including but not limited to cavity preparation processes, the whitening of teeth, laser applications, polishing dental materials, and the polymerization of light-curing materials, may contribute to this phenomenon [2].

Dental resin composite materials have various advantages, including minimal tooth preparation, the adhesion to tooth structures, and esthetic properties. However, the main disadvantages of resin composites are polymerization shrinkage and heat generation during the polymerization reaction. The temperature increase is attributed to two basic factors: light curing and exothermic chain polymerization of the material. Various other aspects must be also considered, including the intensity of the light source, thickness and composition of restorative materials, light-to-material distance, exposure duration, amount of residual dentin, and use of barrier material as a liner or base [3,4].

Considering the chemical formulation of conventional resin composites, almost all used monomers have carbon double bonds (C=C) that are transformed into single bonds (C-C) in an exothermic process to produce linked polymers. A photo-initiator molecule (e.g., camphorquinone/tertiary amine) initiates the exothermic reaction, which is activated by visible blue light using a light-curing unit (LCU) at a wavelength of 380–500 nm and an irradiance >450 mW/cm^2^ [5]. Light-emitting diode (LED) units have replaced quartz–tungsten–halogen (QHT) units. LED LCUs convert electricity to light with near-unity efficiency, reducing heat generation [6]. However, the new generation of LED units produces higher irradiance, thus increasing the risk of pulp damage [7].

Conventional resin-based composites are applied incrementally at a thickness of 2 mm, and the required light irradiation time can vary from 20 s to 40 s for each increment [8]. The incremental technique ensures full depth curing of the placed material for optimal polymerization. In addition, the layering technique minimizes polymerization-shrinkage-induced stress; however, it is a time-consuming and technique-sensitive approach [9,10,11]. Therefore, a bulk-fill resin composite has been introduced to overcome these limitations. Some studies attribute their success to a specific polymerization modulator, improved translucency of the composite resins, and/or the addition of potent initiator systems [12,13]. Compared with conventional resin composites, the bulk-filled type can be applied in thicker increments and polymerized at 4–5 mm of thickness [13]. Light irradiation can reach the full depth and provide sufficient polymerization of the bulk-fill resin composite [14]. However, the sealability of these filling materials have not yet been established [15].

Dental pulp is a highly vascularized tissue that can be subjected to thermal injury during various dental procedures [16]. Zach and Cohen (1965) evaluated the change in pulp temperature in teeth of rhesus monkeys and reported that 15% of the teeth became necrotic when the pulp temperature rose by 5.5 °C above normal body temperature [17]. Another study reported that the risk of thermal injury to the pulp increases with extended radiation exposure and reduced dentin thickness [18].

One of the thermal sensing methods used to measure the heat generated during photo-polymerization of resin composites is the K-type thermocouple wire, which is composed of two dissimilar conductors in contact with one another (nickel-chromium/nickel-aluminum) that produces a voltage when heated. This measures heat at a localized point with direct contact; thus, it is categorized as an invasive technique [19]. Despite this disadvantage, many attempts have been made to evaluate thermal changes during resin composite polymerization using the thermocouple technique [7,20]. An infrared thermal camera (IRT) is a two-dimensional imaging system that is used to screen the temperature rise of an object non-invasively. It has a sensitivity of 0.1 °C and does not require direct contact with the object [21].

After reviewing the literature, it can be identified that limited studies have investigated the effect of the photoactivation of different resin composite thicknesses on pulp chamber temperature in contactless mode. Thus, the aim of this study was to compare the accuracy of the IRT camera and K-type thermocouple thermometer in detecting the elevation in the pulp chamber temperature during resin composite photoactivation using different filling techniques. The null hypotheses were that (I) there were no differences between the IRT camera and K-type thermocouple readings during heat detection and (II) there was no change in the pulp chamber temperature upon resin composite photoactivation, regardless of the filling technique.

## 2. Materials and Methods

### 2.1. Tested Materials

Phosphoric acid etchant (Scotchbond Universal etchant 32%, 3M ESPE, Berlin, Germany) and Scotchbond Universal adhesive (3M ESPE, St. Paul, MN, USA) were used in this study. Two different dental resin composites were used: Filtek Bulk-Fill Flowable Restorative (3M ESPE, Paul, MN, USA) and Filtek Z350XT Flowable Restorative (3M ESPE, Paul, MN, USA). The chemical compositions of the restorative materials are listed in Table 1.

### 2.2. Specimen Preparation

**Figure 1:**
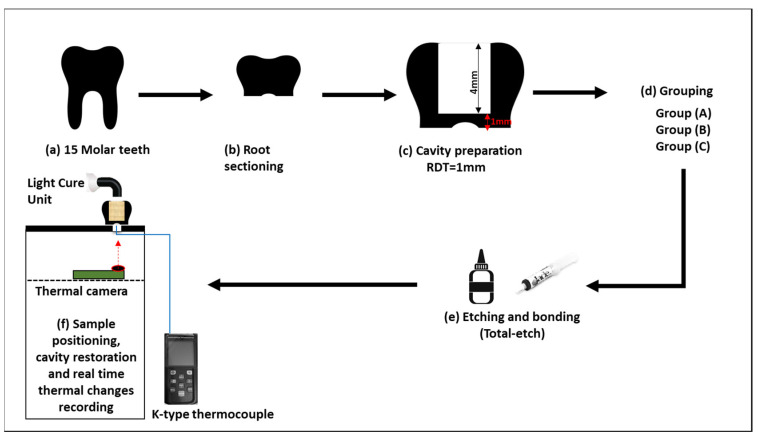
Schematic illustration showing the methodology used in the current investigation.

Fifteen extracted non-carious human permanent molars were used in this study after obtaining ethical approval (195-11-19) from King Abdulziz University, Faculty of Dentistry Institutional Review Board. After cleaning teeth from calculus and tissue debris, the roots of the teeth were sectioned above the furcation area and widened using a large round bur to enable access to the pulp chamber without cutting into the roof of the pulp chamber. Round class I cavity preparations were performed (depth 4 mm × diameter 4 mm) using a large round bur on the occlusal surface of the specimens until the RDT over the pulp from the floor of the cavity was 1 mm. This was measured using a crown gauge caliper placed at the center of the prepared cavity from the occlusal side and the center of the roof of the pulp chamber from the pulpal side. Subsequently, all the samples were divided into three groups (*n* = 5) (A, B, and C; Table 2). A schematic presentation of the methodology of this study is presented in Figure 1.

### 2.3. Heat-Measuring Systems

IRT (Flir) with an accuracy of ±(0.1% of 1 °C) and K-type thermocouple (Perfect Prime TC0520) with an accuracy of ±(0.4% of 0.7 °C) were used to measure the heat generated during the photoactivation of resin composites [21].

**Figure 2:**
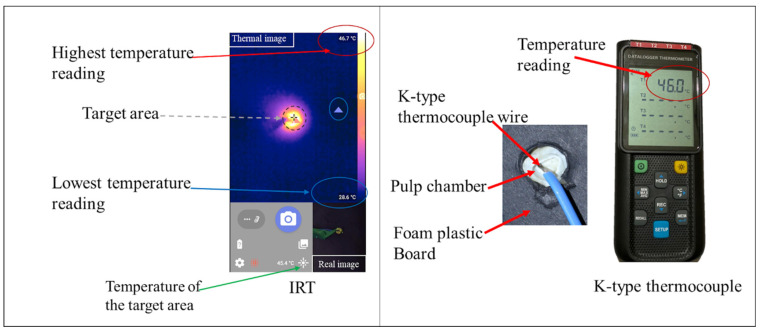
Illustrative figure that shows the main difference between IRT and K-type thermocouple.

Each prepared sample was fixed vertically on a custom-made non-conductive foam plastic board with a hollow opening in the center. Then, the K-type thermocouple wire was fixed to the roof of the pulp chamber through an opening (Figure 2). Simultaneously, the IRT was positioned underneath the specimen, and the camera focus was adjusted for the roof of the pulp chamber (Figure 2). Although both measuring tools have a variable range of temperature measurement, the detected range in this study was set and checked between 0 and 100 °C after pilot testing using ice cubes and boiled water.

### 2.4. Cavity Restoration and Temperature Measurements

Before restoring the prepared cavities, room temperature was set to 25 °C. Each specimen was etched with phosphoric acid and bonded with Scotchbond Universal adhesive in total-etch mode. In group A, cavity restorations were conducted using a Filtek Z350 XT Flowable Restorative composite in three incremental layers with a thickness of 2 mm of the 1st layer followed by 1 mm of the subsequent 2 layers. In group B, cavity restorations were performed using a Filtek Bulk-Fill flowable composite in one bulk increment with a thickness of 4 mm, whereas samples in group C were restored with the Filtek Bulk-Fill Flowable Restorative composite in three incremental layers with a thickness of 2 mm of the 1st layer followed by 1 mm of the subsequent 2 layers. All restorative procedures were performed using an LED LCU (Bluephase N Polywave, Ivoclar Vivadent, 1200–1500 mW/cm^2^). The curing time was 40 s for each layer. The temperature change was monitored and recorded throughout the photoactivation period at every 5 s for each group. The highest temperature during each procedure was concurrently recorded for each device.

### 2.5. Statistical Analysis

The Statistical Package for Social Science software (SPSS for Windows, Version 16.0, SPSS, Chicago, IL, USA) was used to compare the thermocouple and IRT of the same group utilizing independent *t*-test, whereas a one-way ANOVA with a post hoc comparisons test was used to compare the tested groups with either heat-sensing tools at a significance level of α = 0.05.

## 3. Results

**Figure 3:**
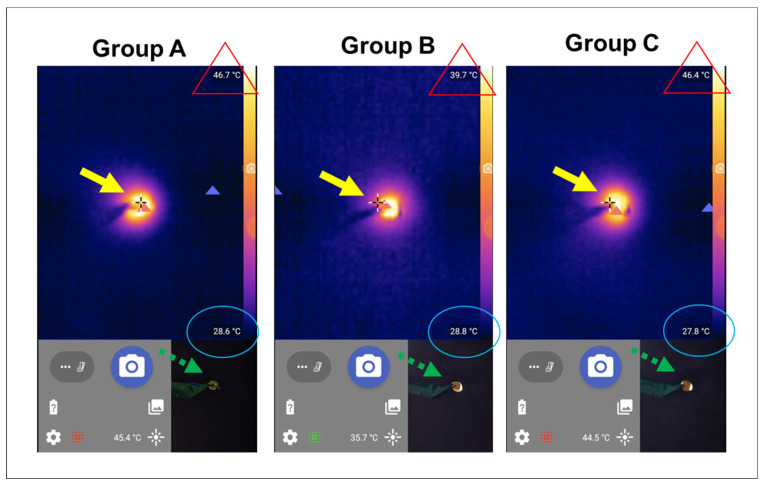
Representative thermal images for the tested groups (**A**–**C**). The red triangle indicates the highest temperature in the image, while the blue oval shapes indicate the lowest temperature in the scanned image. The thermal image shows the target area (pulp chamber) in the center that emits several color pallets, while the dashed green arrow indicates the same target location in the real image.

The time to complete the photopolymerization of all composite increments in each group and the maximum temperature reached by each group were recorded (Figure 3).

**Figure 4:**
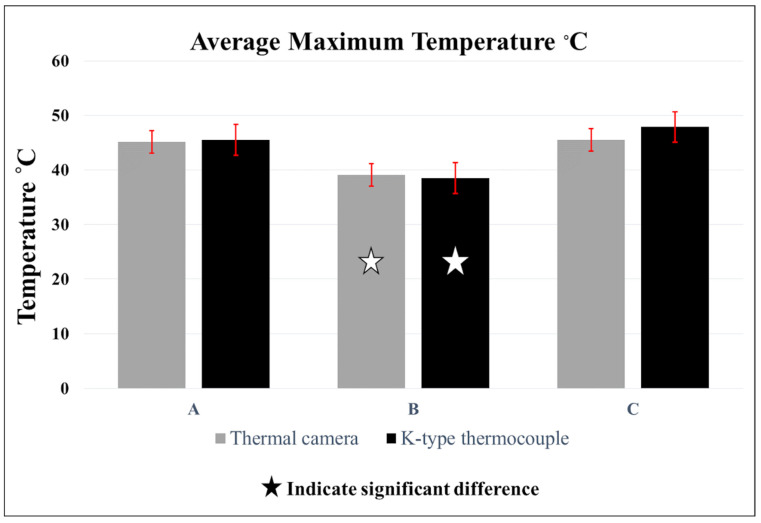
Bar chart showing the average maximum heat temperature in each group (**A**–**C**) using IRT camera and thermocouple measuring tools. One-way ANOVA showed that group (**B**) was significantly different from groups (**A**,**C**) (*p* < 0.05). *T*-test showed no differences within the same group using different temperature-detecting devices (*p* > 0.05).

**Figure 5:**
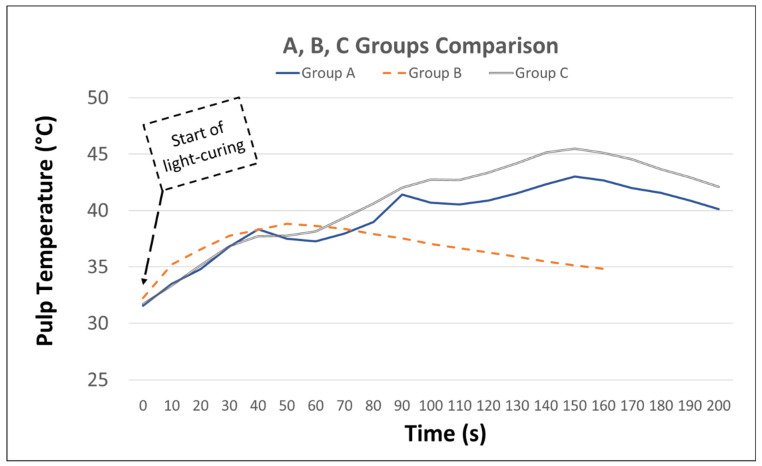
The presented groups (A–C) in this graph show the relation between the curing time (s) and pulp chamber temperature (°C). Repeated light-curing in groups (A, C) showed elevated pulp temperatures over time, unlike group (B).

Table 3 shows the average maximum temperatures recorded by each thermal testing tool within each group (Table 3). An independent *t*-test showed no significant difference between the thermocouple and IRT within the same group (*p* < 0.05). In group A, the average temperatures recorded by the IRT camera and thermocouple were 45.12 ± 2.7 and 45.54 ± 2.7 (*p* = 0.93), respectively, whereas those of group B were 39.14 ± 1.8 and 38.54 ± 2.3 (*p* = 0.69), respectively (Figure 4). Similarly, the results of both thermal sensing tools in group C were comparable (IRT 45.52 ± 2.4 and thermocouple 47.88 ± 1.9) (*p* = 0.16). One-way ANOVA showed that there were significant differences within the groups that had the temperature recorded using the same heat sensing tool (*p* < 0.05). Post hoc analysis further revealed significant differences between group B and group A with both recording devices, the IRT camera (*p* = 0.01) and K-type thermocouple (*p* = 0.003), in which the former group reported lower recorded values. Group B also had lower temperature values compared to group C with the IRT camera (*p* = 0.006) and K-type thermocouple (*p* = 0.003). However, there were no statistical differences between group A and C with either the IRT camera (*p* = 0.97) or K-type thermocouple (*p* = 0.34). Repeated light curing in groups A and C led to an elevation in pulp temperature over time, unlike group B (Figure 5).

## 4. Discussion

Although there are variations in the thermal sensing mechanisms of the K-type thermocouple and IRT camera, the current study findings showed no significant difference between the thermal recording systems within the same group. The K-type thermocouple measures the object temperature in contact mode, whereas the IRT system detects the emitted heat from the polymerized material in contactless mode. Initially, several pilot studies were conducted to determine the best possible recording conditions to obtain a controlled environment without false-positive results. Thus, room temperature was set at 25 °C. In addition, a non-conductive foam plastic board showed better focus of the IRT sensor at the testing site, that is, the pulp chamber, unlike microscope glass slides [22]. Therefore, despite most previous studies using the thermocouple system to measure temperature changes in the pulp chamber, the IRT camera has proven its reliability as a heat-measuring tool [4,19]. This finding agrees with a study by Hamze et al., who concluded that the readings of both thermal sensing systems were comparable [19]. However, the results obtained by Bouillaguet et al. are inconsistent with the current finding as the authors stated that thermocouples may underestimate the heat applied to teeth [7].

Dental resin composite materials consist of inorganic fillers and organic resin matrix. Typical photo-polymerization of these materials results in the activation of a photoinitiator that reacts with double-bonded carbons of the monomer [23]. During this reaction, some highly reactive free radicals are released along with carbon–carbon single bonds that react with another double-bonded carbon monomer. This leads to splitting of the double-bonded carbon and the release of some heat as part of the exothermic reaction [24].

The polymerization of light-activated resin composites results in heat generation during exothermic processes [25]. The increase in temperature caused by various dental procedures has been studied in previous in vivo and in vitro studies in an attempt to increase the safety of these procedures [26,27]. Zach and Cohen et al. studied the pulp temperature changes, and it was reported that 15% of the teeth became necrotic or inflamed when the pulp temperature rose above 5.5 °C; thus, it has been assumed that irreversible damage to the pulp starts at between 42 °C and 42.5 °C [17,28].

The Filtek Bulk-Fill Flowable Restorative is designed for use in deep cavities to reduce the number of restorative steps and improve clinical productivity. According to the manufacturer, it contains fewer monomers and fillers than the Z350 XT Flowable Restorative composite. This semi-translucent material enables a 4 mm depth of cure and contains bis-GMA, UDMA, bis-EMA, TEGDMA, and substituted dimethacrylate. The fillers are silane-treated glass-ceramic and ytterbium trifluoride with particle sizes ranging from 0.01 to 3.5 µm and from 0.1 to 5.0 µm, respectively. The inorganic filler loading is approximately 42.5% by volume. However, the Filtek Z350 XT Flowable Restorative has more monomers and fillers. It contains bis-GMA, TEGDMA, and substituted dimethacrylate. The filler loading is 59.5% by volume and consisted mainly of silane-treated ceramics, silane-treated silica, and ytterbium trifluoride fillers. Although composite ceramic-based fillers have low thermal conductivity in comparison to other fillers [29], composite fillers are more responsible for the thermal conductivity within the material than the composite monomer/polymer [30,31]. However, the authors believe that the variation in the chemical contents of the tested materials has no major influence on the presented findings. Our speculation is supported by the findings of a previous report in 2018, which found that there is no clear influence of resin composite contents on the elevation in dental pulp temperature [19].

Moreover, it was thought that the variation in the composition of the bulk-fill composites would affect the marginal sealing, similar to conventional composites; however, a recent review showed that this variation has no direct influence on this property [15,32,33].

In this study, we examined two filling techniques. The bulk-filling technique is known for faster placement with fewer voids in the mass of the material, which would optimize the chair-side time [34]. An incremental filling technique was introduced to reduce shrinkage stress through resin composite polymerization [8,9,10]. It is inferred from the results that the filling technique has a greater influence on the amount of generated heat than the type of restorative material. Although the amount of heat generated during polymerization is related to the degree of conversion of the contained monomers, polymers, and copolymers, the generated heat is low and dissipates rapidly [19,35]. However, repetitive photoactivation of the composite can lead to a dramatic increase in the cumulative temperature of the irradiated resin composite (Figure 5) [26]. This could explain the elevated heat in the incremental curing of the composites (groups A and C) in comparison with group B (Figure 3) [36]. A previous report found similar results when different composite materials were compared using the incremental filling technique [19].

Within the limitations of this study that include but are not limited to the narrow study design, the evaluation of one type of light curing system, as well as assessment of different resin composite materials at 40 s of curing time, the null hypotheses were partially accepted as there was no significant difference between the two thermal sensing systems, and they were partially rejected as the temperature of the pulp chamber was higher using the incremental filling technique than using the bulk-filling technique. Future studies that include different light-curing units; variable times of light curing, restorative materials, and filling techniques; as well as the quantification of the increase in the pulp temperature in in vivo trials will be considered. Moreover, the use of bulk-fill and conventional resin composites with similar viscosity, consistency, and comparable filler loading needs to be considered in future studies to assess their roles in temperature elevation.

## 5. Conclusions

The IRT camera has excellent potential for non-invasively measuring temperature changes within the material and the dental pulp. Based on the current findings, it appears that increasing the light-curing time and the number of increments has a direct correlation with the elevation of the pulp chamber temperature.

## 6. Highlights

1. The thermal camera is a non-invasive technique that can provide instant data comparable to conventional methods.
2. The elevation in dental composite temperature was mainly related to the filling technique.
3. The chemical composition of the dental composite has no direct influence on heat retention in the material.

## 7. Clinical Application of This Research

Understanding the elevated photopolymerization temperature of dental composites and its potential impact on pulp health integrity is critical for clinical practice in dentistry and has the following clinical applications:

- Material Selection: Dentists can make informed decisions about which composite materials to use for specific cases. For deep or large cavities where there is a higher risk of elevated temperatures, they may opt for materials with lower heat generation during polymerization to minimize the potential harm to the pulp.
- Monitoring: During composite placement, clinicians can monitor the temperature using tools or techniques to ensure it remains within a safe range. This proactive approach allows for adjustments as needed to prevent overheating.
- Patient Education: Dentists can better educate patients about post-operative sensitivity and symptoms to be expected after placing composite restorations. This understanding enables patients to recognize signs of pulp irritation and seek assistance if necessary.
- Research and Development: The information about increased temperature upon polymerization can be used by dental materials manufacturers to develop novel composite materials with superior properties, such as lower heat generation during polymerization to improve patient safety and comfort.

## Figures and Tables

**Table 1:** Chemical composition of the tested materials.

Material	Manufacture (Country)	Composition (% by Wt)
Filtek Z350 XT Flowable Restorative	3M ESPE (USA)	Silane-Treated Ceramic (50–60%) Substituted Dimethacrylate (15–25%) Silane-Treated Silica (5–10%) TEGDMA (<10%) Bis-GMA (1–5%) Ybf3 (1–5%) Poly[oxy(1-oxo-1,6-hexanediyl)], α,α’-(oxydi-2,1-ethanediyl)bis[ω-[[[[2-[(2-methyl-1-oxo-2-propen-1- yl)oxy]ethyl]amino]carbonyl]oxy] (1–5%) N,N-DIMETHYLBENZOCAINE (<0.3%) Diphenyliodonium Hexafluorophosphate (<0.2%) (Filler loading 59.5% Vol)
Filtek Bulk Fill Flowable Restorative	3M ESPE (USA)	Silane-Treated Ceramic (50–60%) Substituted Dimethacrylate (10–20%) UDMA (10–20%) Ybf3 (1–10%) Bis-GMA (1–5%) Bis-EMA-6 (1–5%) TEGDMA (<1%) (Filler loading 42.5% Vol)

Abbreviation: Diurethane Dimethacrylate (UDMA), Bisphenol A Diglycidyl Ether Dimethacrylate (Bis-GMA), Bisphenol A Polyethylene Glycol Diether Dimethacrylate (Bis-EMA-6), Triethylene Glycol Dimethacrylate (TEGDMA), Ytterbium Fluoride (Ybf3).

**Table 2:** Grouping of the tested materials.

Group	Filling Technique
A	Incremental: Filtek Z350 XT Flowable Restorative composite, 3 incremental layers.
B	Bulk: Filtek Bulk Fill Flowable Restorative composite, one layer.
C	Incremental: Filtek Bulk Fill Flowable Restorative composite, 3 incremental layers.

**Table 3:** Comparison of the average maximum temperature between the tested measuring tools in groups A, B, and C. One-way ANOVA showed that group B had significantly lower values compared to groups A and C within each heat-sensing tool (*p* < 0.05). *T*-test showed no differences within the same group using either measuring tools (*p* > 0.05). Same lower-case letters denote no significant difference within the different groups using the same measuring tool, and same upper-case letters denote no significant difference among the same group using different measuring tools.

	Average Maximum Temperature °C (±SD)		
	Group A	Group B	Group C
IRT Camera	45.12 °C (±2.7) ^aA^	39.14 °C (±1.8) ^bA^	45.52 °C (±2.4) ^aA^
Thermocouple	45.54 °C (±2.7) ^aA^	38.54 °C (±2.3) ^bA^	47.88 °C (±1.9) ^aA^

## Data Availability

The data are available upon request.
